# Supplementary material for: Characterisation of Wheat Straw Pellets Individually and in Combination with Cassava Starch or Calcium Carbonate under Various Compaction Conditions: Determination of Pellet Strength and Water Absorption Capacity
Source: Materials (Basel). 2020 Oct 1;13(19):4375. doi: 10.3390/ma13194375 (PMC7579381; doi:10.3390/ma13194375)
Supplement: Supplementary file 1 [file materials-13-04375-s001.pdf]

materials

Article

Supplementary Materials: Characterisation of Wheat Straw Pellets Individually and in Combination with Cassava Starch or Calcium Carbonate under Various Compaction Conditions: Determination of Pellet Strength and Water Absorption Capacity

Patryk Matkowski, Aleksander Lisowski * and Adam Świętochowski

Department of Biosystems Engineering, Institute of Mechanical Engineering, Warsaw University of Life Sciences, Nowoursynowska 166, 02-787 Warsaw, Poland; patryk_matkowski@sggw.edu.pl (P.M.); adam_swietochowski@sggw.edu.pl (A.Ś.)

* Correspondence: aleksander_lisowski@sggw.pl

Table S1. Correlation matrix for type of additive, additive ratio (*A*), material moisture content (*MC*), die height (l_c), material temperature (*t*), DM single pellet density (ρ_P), specific pellet compression work (E_j), elasticity modulus for pellet compression (*E*), pellet tensile strength (σ_c), water absorption by crushed pellets (*k*) and for different types of material.

Type A	Parameters	*A*	*MC*	l_c	*t*	ρ_P	E_j	*E*	σ_c	*k*
WS	*MC*		1.000							
WS	l_c		0.001	1.000						
WS	*t*		0.001	0.001	1.000					
WS	ρ_P		0.124	0.317 [a]	−0.569 [a]	1.000				
WS	E_j		0.252 [a]	0.307 [a]	−0.383 [a]	0.813 [a]	1.000			
WS	*E*		0.255 [a]	0.150	−0.343 [a]	0.711 [a]	0.656 [a]	1.000		
WS	σ_c		0.243 [a]	0.315 [a]	−0.392 [a]	0.891 [a]	0.896 [a]	0.702 [a]	1.000	
WS	*k*		−0.424 [a]	−0.192	0.459 [a]	−0.600 [a]	−0.466 [a]	−0.449 [a]	−0.544 [a]	1.000
WSCS	*A*	1.000								
WSCS	*MC*	0.001	1.000							
WSCS	l_c	0.001	0.001	1.000						
WSCS	*t*	0.001	0.001	0.001	1.000					
WSCS	ρ_p	0.039	0.334 [a]	−0.039	−0.175 [a]	1.000				
WSCS	E_j	−0.042	0.394 [a]	0.051	−0.115	0.514 [a]	1.000			
WSCS	*E*	−0.045	0.290 [a]	−0.109	−0.237 [a]	0.434 [a]	0.446 [a]	1.000		
WSCS	σ_c	−0.058	0.501 [a]	0.004	−0.193 [a]	0.600 [a]	0.830 [a]	0.515 [a]	1.000	
WSCS	*k*	0.009	−0.492 [a]	−0.055	0.157 [a]	−0.502 [a]	−0.450 [a]	−0.240 [a]	−0.505 [a]	1.000
WSCC	*A*	1.000								
WSCC	*MC*	0.001	1.000							
WSCC	l_c	0.001	−0.006	1.000						
WSCC	*t*	0.001	0.006	0.006	1.000					
WSCC	ρ_p	−0.032	0.141 [a]	0.133 [a]	−0.516 [a]	1.000				
WSCC	E_j	−0.022	0.233 [a]	0.039	−0.344 [a]	0.816 [a]	1.000			
WSCC	*E*	−0.101	0.053	0.169 [a]	−0.193 [a]	0.653 [a]	0.623 [a]	1.000		
WSCC	σ_c	−0.026	0.249 [a]	0.088	−0.386 [a]	0.904 [a]	0.928 [a]	0.700 [a]	1.000	
WSCC	*k*	−0.131 [a]	−0.121	−0.174 [a]	0.426 [a]	−0.645 [a]	−0.490 [a]	−0.376 [a]	−0.574 [a]	1.000

[a] statistically significant at p-value = 0.05

Table S2. Regression coefficients of the variables for specific pellet compression work (E_j), elasticity modulus for pellet compression (E), pellet tensile strength (σ_c), water absorption by crushed pellets (k).

	E_j		E		σ_p		k	
	**β*i***	**p-value**	**β*i***	**p-value**	**β*i***	**p-value**	**β*i***	**p-value**
				Wheat straw (WS)				
Intercept	−54.1	<0.0001	−118	<0.0001	−40.2	0.0002	9.39	<0.0001
MC	-	-	-	-	-	-	−0.46	0.0001
l_c	-	-	-	-	0.15	<0.0001	-	-
t	1.23	<0.0001	2.72	<0.0001	0.79	0.0008	-	-
MC^2	-	-	-0.01	0.0001	-	-	0.007	0.0019
l_c^2	0.001	<0.0001	-	-	-	-	-	-
t^2	−0.007	<0.0001	−0.02	<0.0001	−0.005	0.0001	-	-
$MC \times l_c$	−0.004	0.0010	-	-	−.003	0.0027	−0.002	0.0030
$MC \times t$	0.004	0.0001	0.006	<0.0001	0.004	0.0004	0.003	<0.0001
$l_c \times t$	-	-	0.0007	0.0075	-	-	-	-
F test	76.6		115		94.2		510	
R	0.7288		0.7614		0.6809		0.7276	
				Wheat straw + cassava starch (WS+CS)				
Intercept	1.08	<0.0001	-	-	−22.8	0.0089	5.77	<0.0001
A	-	-	-	-	-	-	0.11	0.0306
MC	-	-	-	-	-	-	−035	<0.0001
l_c	-	-	-	-	0.64	0.0057	-	-
t	-	-	0.24	<0.0001	-	-	-	-
A^2	-	-	-	-	-	-	−0.009	0.0302
MC^2	0.003	<0.0001	0.02	<0.0001	0.005	<0.0001	-	-
l_c^2	-	-	-	-	−0.004	0.0058	−0.001	<0.0001
t^2	-	-	−0.001	<0.0001	-	-	−0.0004	0.0007
$A \times MC$	-	-	-	-	-	-	-	-
$A \times l_c$	-	-	0.01	0.0050	-	-	-	-
$A \times t$	-	-	−0.01	0.0034	-	-	-	-
$MC \times l_c$	-	-	−0.007	<0.0001	-	-	0.003	<0.0001
$MC \times t$	−0.0007	0.0098	-	-	-0.001	<0.001	0.0009	0.0048
$l_c \times t$	-	-	-	-	-	-	0.0009	0.0022
F test	118		2382		87.7		569	
R	0.4347		0.4831		0.6116		0.6508	
				Wheat straw + calcium carbonate (WS+CC)				
Intercept	-	-	−86.8	<0.0001	-	-	44.0	<0.0001
A	−1.24	0.0021	−0.94	0.0002	−0.23	0.0136	-	-
MC	-	-	-	-	-	-	−0.79	<0.0001
l_c	-	-	1.36	0.0001	-	-	-	-
t	0.24	<0.0001	0.89	<0.0001	0.14	<0.0001	−0.72	<0.0001
A^2	-	-	-	-	-	-	−0.006	0.0018
MC^2	−0.02	<0.0001	−0.006	0.0010	−0.01	<0.0001	0.01	<0.0001
l_c^2	-	-	−0.006	0.0039	-	-	−0.0002	0.0002
t^2	−0.003	<0.0001	−0.004	<0.0001	−0.002	<0.0001	0.004	<0.0001
$A \times MC$	0.02	0.0016	0.009	0.0188	0.01	0.0145	-	-
$A \times l_c$	-	-	-	-	-	-	-	-
$A \times t$	0.009	0.0290	0.008	0.0028	-	-	-	-
$MC \times l_c$	-	-	−0.004	0.0021	-	-	-	-
$MC \times t$	0.007	<0.0001	0.005	<0.0001	0.006	<0.0001	0.002	0.0025
$l_c \times t$	-	-	−0.003	0.0013	-	-	-	-
F test	359		57.0		77.7		494	
R	0.5833		0.5895		0.6099		0.6956	
				For all				
Intercept	−20.6	0.0045	−46.8	0.0001	−16.9	0.0025	6.92	<0.0001
A	−0.18	0.0048	-	-	-	-	−0.42	<0.0001
MC	-	-	0.08	<0.0001	-	-	−0.30	<0.0001

l_c	0.024	0.0276	-	-	0.02	0.0052	-	-
t	0.52	0.0010	1.15	<0.0001	0.41	0.0008	-	-
A^2	-	-	-	-	-	-	0.03	0.0001
MC^2	−0.006	<0.0001	-	-	−0.003	0.0080	0.007	<0.0001
l_c^2	-	-	-	-	-	-	-	-
t^2	−0.003	0.0001	−0.007	<0.0001	−0.003	<0.0001	0.0003	<0.0001
$A \times MC$	0.007	0.0232	-	-	-	-	-	-
$A \times l_c$	-	-	-	-	-	-	-	-
$A \times t$	-	-	-	-	-	-	-	-
$MC \times l_c$	-	-	-	-	-	-	-	-
$MC \times t$	0.003	<0.0001	-	-	0.002	<0.0001	-	-
$l_c \times t$	-	-	-	-	-	-	−0.0002	0.0107
F test	86.1		270		135		432	
R	0.4484		0.3078		0.4909		0.4536	

A: additive ratio, MC: moisture content, l_c: die height, t: material temperature, R: goodness of fit.

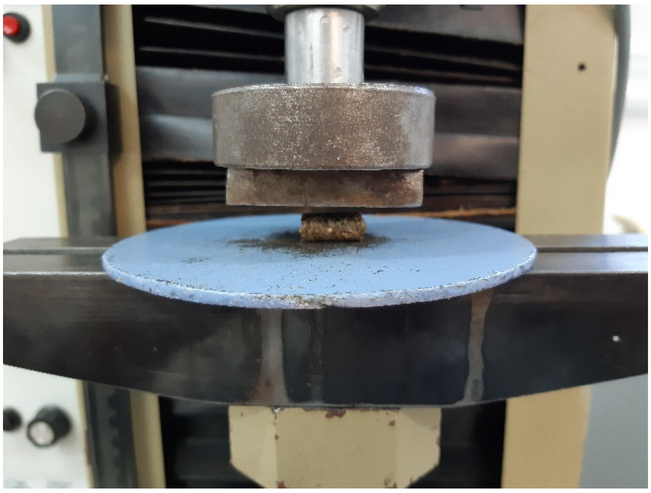

Figure S1. Pellet compression on the universal testing machine TIRAtest.

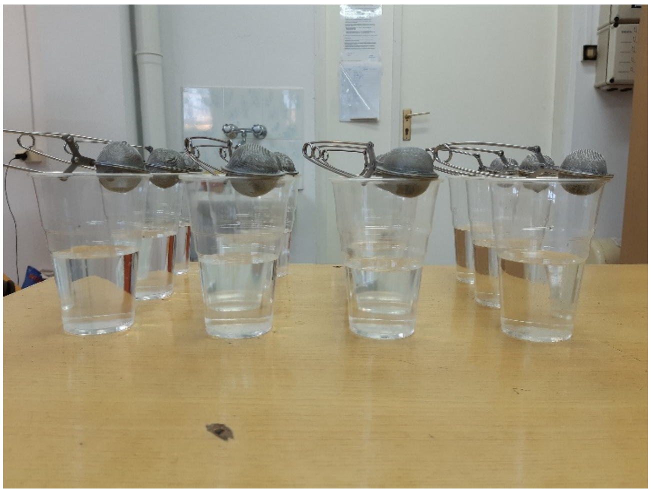

Figure S2. Water absorption test for crushed pellets from the WS blend with the addition of CS.
